# The Development of an AIDS Mucosal Vaccine

**DOI:** 10.3390/v2010283

**Published:** 2010-01-22

**Authors:** Xian Tang, Zhiwei Chen

**Affiliations:** 1 AIDS Institute, Li Ka Shing Faculty of Medicine, The University of Hong Kong, Hong Kong SAR, China; 2 Department of Microbiology and Research Center of Infection and Immunology, Li Ka Shing Faculty of Medicine, The University of Hong Kong, Hong Kong SAR, China

**Keywords:** HIV-1, AIDS, vaccine, mucosal vaccination, MVTT

## Abstract

It is well known that mucosal tissues contain the largest surface area of the human body and are the front line of natural host defense against various pathogens. In fact, more than 80% of infectious disease pathogens probably gain entry into the susceptible human hosts through open mucosal surfaces. Human immunodeficiency virus type one (HIV-1), a mainly sexually transmitted virus, also primarily targets the vaginal and gastrointestinal mucosa as entry sites for viral transmission, seeding, replication and amplification. Since HIV-1 establishes its early replication in vaginal or rectal mucosal tissues, the induction of sufficient mucosal immunity at the initial site of HIV-1 transmission becomes essential for a protective vaccine. However, despite the fact that current conventional vaccine strategies have remained unsuccessful in preventing HIV-1 infection, sufficient financial support and resources have yet to be given to develop a vaccine able to elicit protective mucosal immunity against sexual transmissions. Interestingly, Chinese ancestors invented variolation through intranasal administration about one thousand years ago, which led to the discovery of a successful smallpox vaccine and the final eradication of the disease. It is the hope for all mankind that the development of a mucosal AIDS vaccine will ultimately help control the AIDS pandemic. In order to discover an effective mucosal AIDS vaccine, it is necessary to have a deep understanding of mucosal immunology and to test various mucosal vaccination strategies.

## 1. Introduction

Mucosal membranes contain the largest surface area of the human body, providing protection for various body cavities exposed to the external environment and internal organs. Mucosal membranes comprise the linings of gastrointestinal, urogenital and respiratory tracts [1]. They are actually the portal of entry for more than 80% of infectious disease pathogens, and the first line of defense for host protection from infection through various mechanisms including physical barriers, mechanical discharge, chemical degradation, innate immunity, and specialized mucosal immune responses [2,3].

Mucosal tissues represent a critical immunological repertoire, which is compartmentalized and independent from systemic immune organs [4]. Mucosal tissues in a healthy human body comprise as much as 80% of all lymphocytes, which may transfer between various mucosal-associated lymphoid tissues (MALTs) [4]. B-lymphocytes in MALTs mainly produce secretory IgA or sIgA, which is released as a covalently linked dimer. This unique structure makes sIgA more stable and resistant to many kinds of digestive enzymes. sIgA is considered to be the major protective immunoglobulin on mucosal surfaces [4]. T lymphocytes on the other hand either act as cytotoxic T lymphocytes (CTL) (e.g., CD8^+^ T cells) or help the induction of both cellular mediated immunity and antibody response (e.g., CD4^+^ T cells) [4].

Upon encountering foreign antigens, the activated B and T cells may migrate from inductive sites (e.g., Peyer’s patches in the gut, and nasopharynx-associated lymphoid tissue (NALT) in the oropharyngeal cavity), travel through lymph nodes and the peripheral circulation and back home to effector sites (e.g., such as the lamina propria of the intestinal and respiratory tracts, and glandular tissues). This results in the generation of antigen-specific T helper 2 (Th2) cell-dependent IgA responses, and T helper 1 (Th1) cell- and CTL-dependent immune responses, which function as the front line of defense at mucosal surfaces. The homing behavior of these cells is mainly attributed to the up-regulated expression of site-specific adhesion molecules and cytokine receptors that guide the cells back to mucosa by recognition of mucosal tissue-specific receptors on vascular endothelial cells [5,6]. Critically, mucosal inductive sites stand as sentinels to the intestinal and respiratory systems and represent the major sites where mucosal immune responses are initiated.

Human immunodeficiency virus type one (HIV-1), the causative agent of acquired immunodeficiency syndrome (AIDS), is a mainly sexually transmitted virus. HIV-1 primarily targets the gastrointestinal and vaginal mucosa as entry sites for viral transmission, seeding, replication and amplification. Since HIV-1 establishes its early replication in vaginal or rectal mucosal tissues, the induction of sufficient mucosal immunity at the initial site of HIV-1 transmission becomes essential for a protective vaccine. Here, we provide an overview of the development of an AIDS mucosal vaccine and perspectives of mucosal vaccine candidates against HIV-1.

## 2. A Brief History of Mucosal Vaccines

A vaccine is a biological preparation that induces immunity against a particular disease. The term vaccine derives from Edward Jenner's use of the term cowpox (Latin *variolæ vaccinæ*, adapted from the Latin *vaccīn-us*, from *vacca* cow) in 1796. Edward Jenner demonstrated that cowpox, when administered to humans, provided them protection against smallpox. The history of using a biological preparation to prevent smallpox, however, was traced back to Northern Song Dynasty (960–1127 A.D.) when Chinese Ancestors collected scabs from skin lesions of smallpox patients and used the ground down particles to prevent new infections, a procedure called variolation [7]. Interestingly, the procedure involved the delivery of the biological preparation through intranasal inoculation, a common route of modern mucosal vaccination. This technique was subsequently introduced to European countries by Lady Mary Montagu prior to 1762, which later led to the discovery of vaccines and modern immunology [8].

During recent decades, with the rapid development of modern immunology, significant progress has been made to understand the structure and function of the mucosal immune system. It is now clear that mucosal vaccines educate immune cells in mucosal inductive sites and can thus induce protective immunity in both the mucosal and systemic compartments. One of the classical mucosal vaccines is the oral polio vaccine (OPV), a live-attenuated strain with spontaneous mutations in the viral genome. OPV was generated by a series of passages of a polio virus through non-human cells at sub-physiological temperature [9]. In comparison to the inactive polio vaccine (IPV) given by injection, OPV not only elicits neutralizing antibodies to prevent the spread of poliovirus to the nervous system, but also induces excellent sIgA response in the intestinal mucosa, the primary site of poliovirus entry and replication, which contributes greatly to the worldwide eradication of polio. However, because of concerns over the reversion of the live-attenuated vaccine into a virulent form, OPV has been largely replaced by IPV in developed countries.

Another well known mucosal vaccine in recent years is FluMist®, which is a live attenuated, trivalent cold-adapted influenza vaccine (CAIV-T) manufactured by MedImmune, Inc [10–12]. FluMist is given as a gentle nasal mist. This nasal vaccine has been proved to be safe, effective and well tolerated in adults including those infected with HIV-1. It was approved by the United States Food and Drug Administration (FDA) in 2003 and became available on the market in the same year [10–12]. The current version of CAIV-T is a refrigerator-stable formulation of FluMist®, containing components of live attenuated influenza viruses of each of the three strains for the 2009–2010 season: A/South Dakota/6/2007(H1N1) (an A/Brisbane/59/2007-like), A/Uruguay/716/2007 (H3N2) (an A/Brisbane/10/2007-like), and B/Brisbane/60/2008. These findings have demonstrated the successful application of mucosal vaccines in protecting humans from viral infections.

## 3. HIV-1 infection in mucosal associated lymph tissues

Since conventional vaccines have not proven successful against HIV-1 infections, it is necessary to explore other strategies including mucosal vaccination. Genetically divergent HIV-1 and its cousin, simian immunodeficiency virus (SIV), use host proteins CD4 and the chemokine receptor CCR5 as primary and secondary receptors [13,14]. Moreover, HIV-1 and SIV both replicate optimally in activated memory CD4^+^CCR5^+^ T cells, a cell type that is abundant in mucosal lymph tissues [15]. It was first demonstrated in the SIV/macaque model system that the mucosal lymph tissues are the initial and predominant sites of SIV infection [15,16]. At the acute stage of infection, not only was the high viral load detected in the intestinal mucosa, but SIV infection resulted in pathogenic effects as indicated by the depletion of a significant proportion of CD4^+^ T cells in the guts but not in peripheral blood [17].

Since at this stage, similar effects have not been found in peripheral lymphoid tissues, the intestine appears to be a major target for SIV replication and the major site of CD4^+^ T cell loss in early SIV infection. In fact, the gut-associated lymph tissues contain about 40% of the total lymphocytes [18]. Similar findings were subsequently made in macaques infected with human/simian immunodeficiency virus (SHIV) harboring CCR5-tropic HIV-1 envelope, implicating the mucosal lymph tissues as being the initial and predominant sites of HIV-1 infection [19,20]. CD4^+^ T cell loss predominates in the effecter sub-compartment of the gastrointestinal (GI) mucosa at all stages of HIV-1 disease [21]. This loss did not appear to be easily reversible as there was a significantly greater CD4^+^ T cell loss in the GI mucosa despite over five years of fully suppressive therapy [21,22].

Since sexually transmitted HIV-1 establishes its initial replication in mucosal tissues, the induction of sufficient mucosal immunity at the initial site of HIV-1 transmission becomes essential for the development of a protective vaccine [16,18]. Some studies have tried to understand the early events following the transmission of SIV or HIV-1 via the mucosal surfaces. Both viruses were found to replicate predominantly in CD4^+^ T cells at the portal of entry and in lymphoid tissues [23]. The viruses were found propagating not only in activated and proliferating T cells but also in resting T cells. Because most of the HIV-1-infected resting T cells persisted after antiretroviral therapy, it poses great difficulties when attempting to eradicate the virus *in vivo* [23]. However, SIV did not readily disseminate to the systemic circulation but rather localized to the mucosa of viral inoculation at the very early stage of infection [23,24]. Moreover, the mucosal barrier greatly limits the infection of cervicovaginal tissues, and thus the initial founder populations of infected cells are smaller [25,26]. Recent findings have further indicated that a dramatic evolutionary bottleneck occurs, with 80% of heterosexual infections apparently initiated by a single variant when HIV-1 is transmitted into a new recipient [27,28]. The initially small founder populations and dependence on continuous seeding, to establish a productive infection in systemic lymph tissues, define a small window of maximum vulnerability for the virus in which there is an opportunity for the host, vaccines, or other interventions to prevent or control infection [26]. The depletion of memory CD4^+^ T cells, especially in the gut during the acute phase of infection, provide further evidence that the induction of mucosal immunity should be considered a high priority in the development of vaccines against mucosally transmitted HIV-1 [18,29,30]. These findings have provided supporting evidence for strategies by focusing on mucosal vaccines for inducing protective HIV-1 immunity at the site of viral transmission.

## 4. Protective mucosal immune responses at the site of viral transmission

One of the critical bottlenecks in the development of an effective HIV-1 vaccine is the current inability to induce a local mucosal immunity with a durable, high level of antibody response and high frequency cellular immune response in humans. Mucosal sIgA is considered to have an important role in the prevention of HIV-1 transmission through sexual intercourse [31]. Several studies have evaluated the neutralizing activity of sIgA purified from plasma and mucosal samples from HIV-1 highly exposed persistently seronegative individuals (HEPSIs) or infected people [32–34]. Against HIV-1 primary isolates of different viral clades and phenotypes, specific neutralizing activity of the purified IgA from cervicovaginal fluid was found in the majority of samples [32–34]. In contrast, the cervicovaginal fluid of low-risk, uninfected HIV-seronegative individuals lacked neutralizing IgA [32,33]. Moreover, mucosal HIV-1 gp41-specific IgA derived from highly exposed seronegative individuals was able to block HIV-1 epithelial transcytosis and neutralized CD4^+^ cell infection [35]. However, by analyzing a group of exposed uninfected female commercial sex workers from Gambia, no significant vaginal sIgA or IgG responses against HIV-1 or HIV-2 were detected, and none of the vaginal secretions tested displayed any HIV-1 neutralizing activity [36,37]. Since these studies seem controversial, it becomes necessary to study neutralizing IgA or IgG induced by a mucosal vaccine [31]. Interestingly, a recent study indicated that anti-CCR5 IgG and IgA might have contributed to protection against HIV-1 sexual transmission among exposed seronegative people [38]. It is known that mucosal polymeric IgA plays a predominant role in the protection of influenza virus-induced pathology in the upper respiratory tract [39]. Whether or not similar effects can be achieved against HIV-1 remain unknown. It was evident that functional polymeric IgA-like 2F5, by switching 2F5IgG to the IgA isotype, had a stronger impact on the protective potential of these two antibodies and could interfere with HIV-1 entry across a mucosal epithelial layer *in vitro* [40]. These findings indicate that although sIgA is the major humoral defense mechanism at mucosal surfaces, it is wise to induce locally produced IgM and IgG as well as serum-derived IgG via mucosal vaccination. Mucosal neutralizing IgG is also important because it protects macaques from repeated intravaginal exposure to low doses of a SHIV that uses CCR5 as its co-receptor [41,42].

It has been demonstrated in SIV/macaque models that CD8^+^ cytotoxic T lymphocytes (CTL) play a crucial role in suppressing SIV replication *in vivo* during acute and chronic infections [43,44]. In fact, based on a mathematical model, it has been suggested that the first T cell response to transmitted/founder viruses in humans is early and contributes to the initial decline of plasma virus in acute infection [45]. Besides mucosal antibody response, high frequency mucosal cellular immune responses detected are essential for preventing the local productive infection and eliminating the initial founder populations of infected cells [45–49]. HIV-1-specific CD8^+^ T cell responses were found in the genital mucosa of HIV-1-resistant sex workers in the absence of detectable HIV-1 infection, suggesting the protective role of cell-mediated immunity [50]. Moreover, cellular immune responses, especially those mediated by CTL and CD4^+^ helper T lymphocytes, are needed to control HIV-1 or SIV in the mucosal tissues [46–48,51]. In particular, the magnitude and timing of the establishment of an excess of effector cells *versus* targets were found to correlate with the extent of control and SIV infection outcome [16]. Therefore, vaccines capable of inducing high frequency broadly reactive cell-mediated responses are considered critical for controlling the spread of the virus [45,52]. Although multiple approaches have been developed for inducing mucosal CTL responses, these CTLs were weak or at low frequency and could not completely prevent the initial viral replication and dissemination [46–48,53].

## 5. Vector-based AIDS mucosal vaccine strategies

To develop an effective HIV-1 mucosal vaccine, besides immunogen design, there are three factors to be considered: the vector for antigen delivery, the adjuvant to enhance mucosal immune response and the route of immunization to induce both mucosal and systemic immune responses as early as possible [54–57]. The selection of a proper antigen delivery vector is necessary to the induction of an efficient mucosal immune response. Studies in macaques found that immunization with a live attenuated SIV vaccine can induce specific local CTLs in mucosa and dramatically reduce viremia after mucosal challenge with SIV [58–60]. Since a live attenuated HIV-1 is too risky to be used as a human vaccine, other viral vectors are under investigation for stimulating mucosal immunity, such as live attenuated vesicular stomatitis virus, non-replicating adenovirus, vaccinia virus including modified vaccinia Ankara (MVA) vectors, and Venezuelan equine encephalitis virus (VEE). To this end, the vaccinia viral vector is probably one of the most intensively studied live recombinant vectors.

Several studies have demonstrated the ability of vaccinia-based vaccines in inducing mucosal immune responses against infectious pathogens [61–63]. For example, either mucosal or systemic routes of immunization with the live, attenuated vaccinia NYVAC/SIVgpe recombinant vaccine resulted in gag-specific CD8^+^ T-cell responses in mucosal tissues of macaques [49]. Furthermore, vaccines based on the MVA vector were effective in inducing protective responses against different respiratory viruses such as SARS-CoV, influenza and respiratory syncytial virus following immunization via mucosal routes [64–70]. Intranasal inoculation of the MVA-based HIV-1 vaccine (named MVA-HIV) was immunogenic, whereas the intravaginal route was disappointing [62]. Since the mucosal immunogenicity of MVA-HIV is low, new approaches are needed to improve the poxvirus vector system [62]. Several approaches have been evaluated in animals. First, intranasal co-delivery of MVA-HIV plus adjuvant cholera toxin (CT) significantly enhanced the cellular and humoral immune response against HIV-1 antigens at the mucosal surfaces of vaccinated mice. Due to the limitation of CT for human use, cytokines IL-1alpha, IL-12, IL-18 and GM-CSF have been evaluated as substitutes for the induction of systemic and mucosal CTL after nasal immunization [71]. Thus, the functional activity of these candidate adjuvants needs to be tested in the context of MVA-HIV. Second, a heterologous DNA-HIV prime-MVA-HIV boost by intranasal immunization, together with CT, produced a cellular immune response in the mouse spleen 10-fold superior to that in the absence of CT [62]. Moreover, in a study using rhesus macaques, intranasal heterologous vaccination with SHIV-DNA plus IL-2/Ig DNA and rMVA induced both systematic and mucosal immunity and protected animals from disease progression against the intrarectal challenge with pathogenic SHIV89.6P [72]. In a separate study, rhesus macaques vaccinated intrarectally with a DNA construct producing replication-defective SHIV particles and boosted with rMVA-SHIV produced virus-specific mucosal and systemic humoral IgA and cell-mediated immune responses [63]. This mucosal vaccination regimen was also sufficient to delay progression to AIDS [63,73]. Although these findings are promising, there seem to be limited advantages for vaccination via the mucosal routes in comparison to the non-mucosal route of vaccination. For example, intramuscular inoculation of a heterologous DNA/MVA vaccine regimen showed a similar level of protection in monkeys [74]. It remains unknown whether or not a much better protection could be achieved after vaccinia, or other vector systems, are further optimized for stimulating host mucosal immune responses.

## 6. New strategies for the development of an HIV-1 mucosal vaccine

A variety of novel biological vehicles have been studied for the delivery of HIV-1 antigens including inert system and live recombinant vector system [3]. The inert system involves the direct incorporation of HIV-1 antigens into a vehicle in order to increase their immunogenicity, to protect them from degradation, and to enhance their uptake by mucosal surfaces. Commonly used inert vectors include liposomes, immunostimulating complexs (ISCOMs), lactic acid and lectins. Most of these vectors, however, are still at the early stage of preclinical studies. Since these vectors can be easily manipulated *in vitro*, the targeted delivery of HIV-1 antigens into antigen presentation cells (APC) might help improve the immunogenicity of mucosal vaccines of these types. For the live recombinant vector system, a number of studies have been carried out using bacterial vectors encoding HIV-1 antigens. In a phase I study, the safety and immunogenicity of an oral attenuated Salmonella enterica serovar Typhimurium delivering an HIV-1 Gag antigen via the Salmonella Type III secretion system was investigated among healthy volunteers [75]. Although more than 80% of the subjects had mucosal immune responses to vector antigens, responses specific to HIV-1 Gag were low and disappointing. Since some vaccines in the high dose group developed severe adverse events, such as diarrhea, fever, and abnormal transaminases, the vaccine probably requires further attenuation and laboratory research [76].

We have recently reported a novel replication-competent modified vaccina Tian-Tan (MVTT) as a promising mucosal vaccination vector [77]. Using SARS-CoV spike glycoprotein as a test antigen, we conducted a head-to-head comparison of MVA-S and MVTT-S. After these two vaccines were inoculated into mice via seven different routes of immunization, we found that the neutralizing antibody (Nab) response induced was significantly different between MVTT-S and MVA-S. First, intranasal (i.n.) and intraoral (i.o.) routes clearly induced the highest level of Nabs for MVTT-S but not for MVA-S. Second, if we compare the levels of Nabs elicited via the i.n. and i.o. immunizations, MVTT-S induced more than 100-fold higher Nab response than MVA-S did in this experiment. Moreover, we increased the inoculation dose of MVA-S by 10-fold (10^6^ pfu/mouse) and did not observe significant improvement in the Nab response in mice via either i.o. or i.n. routes. In contrast, 10^4^ pfu of MVTT-S was still able to induce significant levels of Nab response in mice via the same i.n. and i.o. routes. Therefore, our data indicate that MVTT-S may preferentially target the mucosa immune system, signifying its superiority to the MVA vector system. Further studies will evaluate MVTT as a mucosal AIDS vaccine using an SIV/macaque model. Although some animal investigations have led to the hypothesis that systemic vaccines can induce virus-specific CTLs at mucosal sites [48,78,79], it remains plausible that mucosal vaccination remains the most effective way for inducing high and long-term protective immunity at the mucosal sites of viral transmission including HIV-1 [80].

## 7. Summary and perspectives

With the increasing efforts in understanding the mucosal transmission of HIV-1, a greater attention has been drawn to study AIDS mucosal vaccines. Since conventional vaccine strategies have not been successful for use against AIDS, it is necessary to provide sufficient financial support and resources to study protective mucosal immunity and new vaccine methods against HIV-1 sexual transmissions. Up till now, there are still many unanswered questions for the development of a protective HIV-1 mucosal vaccine. These unanswered questions are related to HIV-1 diversity in mucosal tissues, the role of innate mucosal immunity, the lack of understanding of immune correlates of mucosal protection, the rapid formation of a latently infected CD4^+^ T-cell pool in mucosal reservoirs, the depletion of CD4^+^ T cells especially in guts through all stages of infection, the absence of effective mucosal adjuvants for human use and the poor immunogenicity of current envelope antigens to induce broadly neutralizing anti-HIV antibodies at mucosal surfaces.

The failure of Merck’s rAd5-based HIV-1 vaccine has called for efforts to further study HIV-1 immunology and vaccinology. It has been evident that it is possible to identify cell-mediated correlates of protection when the vaccine regimen is strong enough. An encouraging finding came from a recent study where the heterologous rAD26SIVgag/rAD5SIVgag regimen elicited strong cell-mediated immune responses, which correlated with significant immune control of a pathogenic SIV challenge [81]. To this end, the evaluation of various heterologous prime and boost vaccine regimens should be studied especially through mucosal routes of vaccination. This is a critical area of investigation because the magnitude and timing of the establishment of an excess of effector cells *versus* targets were found to be essential to the mucosal control of SIV infection [16].

Recent news from the U.S. Military HIV Research Program and the Thai Ministry of Public Health indicated that a prime-boost combination of two AIDS vaccine candidates has shown partial efficacy. Those administered with the combination vaccine has a 31% increased chance of preventing infection with HIV-1, as shown in a phase III efficacy trial in Thailand when the ‘intent-to-treat’ strategy of data analysis was modified [82]. When data were analyzed based on ‘per-protocol’ or ‘intent-to-treat’ analyses, differences dropped to 26% and results did not reach statistical significance [82]. Although it remains unknown whether or not vaccine-induced immunity has contributed to the partial protection at this stage, this finding is the first demonstration that a candidate AIDS vaccine provides benefit in humans. The AIDS vaccine research and development remain a long march. In order to discover an effective mucosal AIDS vaccine, it is necessary to have a deep understanding of mucosal immunology and to test various vaccine strategies including mucosal vaccination, which follows the fundamental rules of host natural defense.
